# Genomic modeling of hepatitis B virus integration frequency in the human genome

**DOI:** 10.1371/journal.pone.0220376

**Published:** 2019-07-29

**Authors:** Ondrej Podlaha, George Wu, Bryan Downie, Raghuraman Ramamurthy, Anuj Gaggar, Mani Subramanian, Zhishen Ye, Zhaoshi Jiang

**Affiliations:** Gilead Sciences Inc., Foster City, CA, United States of America; Centre de Recherche en Cancerologie de Lyon, FRANCE

## Abstract

Hepatitis B infection is a world-wide public health burden causing serious liver complications. Previous studies suggest that hepatitis B integration into the human genome plays a crucial role in triggering oncogenic process and may also constitutively produce viral antigens. Despite the progress in HBV biology and sequencing technology, our fundamental understanding of how many hepatocytes in the liver actually carry viral integrations is still lacking. Herein we provide evidence that the HBV virus integrates with a lower-bound frequency of 0.84 per diploid genome in hepatitis B positive hepatocellular cancer patients. Moreover, we calculate that integrated viral DNA generates ~80% of the HBsAg transcripts in these patients. These results underscore the need to re-evaluate the clinical end-point and treatment strategies for chronic hepatitis B patients.

## Introduction

Hepatitis B (HBV) infection is one of the leading causes of liver fibrosis, liver function failure, and hepatocellular carcinoma worldwide [1–6]. The virus has a compact circular genome comprised of 3.2Kb partially double-stranded DNA, which encodes four overlapping genes essential for the regulation, replication, and the formation of the nucleocapsid and the viral envelope [4–6]. During the HBV replication cycle, failure of the template-switching process results in the formation of a linear double-stranded HBV DNA [7], which can then integrate into the human genome. Although current anti-viral treatments can significantly suppress HBV replication, HBsAg seroconversion, the most widely applied clinical endpoint for evaluating HBV treatment success, is rarely achieved in chronic hepatitis B (CHB) patients [8]. A recent RNAi-based treatment study [9] demonstrated that integrated HBV DNA can contribute significantly to serum HBsAg expression. However, this conclusion was derived from a small number of chronically infected chimpanzees. It is important to confirm this observation in a large cohort of CHB patients. In order to better understand the magnitude and clinical relevance of HBV integration in CHB subjects, we set out to quantify: i) the frequency of independent HBV integration events and ii) the fraction of viral HBsAg transcription originating from integrated HBV DNA. Due to the invasive nature of liver biopsy, it is challenging to obtain genomic datasets for large CHB patient cohorts. In contrast, whole genome and transcriptome studies from surgically dissected HBV infected hepatocellular carcinoma (HCC) samples are widely available, including adjacent non-tumor liver tissue [10–14]. Although the adjacent non-tumor liver tissue samples from HCC patients may not exactly resemble the genetic and transcriptional profile of non-HCC CHB patients, it serves as the most relevant surrogate sample for this data analysis. We therefore collected whole genome and transcriptome sequencing data from two large genomic studies of HCC patients [10, 14] and estimated the frequency of HBV DNA integration in non-tumor liver samples and the fraction of HBsAg expression from integrated HBV DNA copies.

## Materials and methods

### Dataset

We assembled liver cancer dataset from Fujimoto et al. [15] and Sung et al. [14], which included DNA and RNA sequencing data from tumor and adjacent non-tumor liver tissues. Our analysis focused only on a subset of HBV positive HCC samples defined by the detection of HBV DNA or RNA reads from sequencing data. The final dataset contained a total of 192 DNAseq samples (120 tumor and 72 adjacent non-tumor samples) and 60 RNAseq samples (30 tumor and 30 adjacent non-tumor samples S1 Table). Majority of these samples were paired tumor and non-tumor adjacent samples. Datasets were downloaded from the European Genome-phenome Archive https://www.ebi.ac.uk/ega, accession numbers EGAD00001001881, EGAD00001001880, and PRJEB2869. Clinical information related to serum HBeAg status, HBsAg levels, ALT, and other viral biomarkers were not publically available. HBV genotype was inferred via viral read mapping (see below).

### Identification of HBV integrations

RNAseq and DNAseq read pairs were first aligned using bwa-mem[16] to the reference genome of HBV genotypes A, B, C, D, E, F, and H. HBV genotype for each sample was determined by assessing which HBV reference genotype produced the highest quality alignment. From here on, each sample was then processed using the best matching HBV genotype. Pairs of reads in which at least a portion of one read mapped to the HBV genome were retained and re-aligned to a hybrid GRCh38:HBV genome reference sequence in which the HBV sequence was included as a pseudo-chromosome (GenBank: GQ924620). In order to quantify viral insertion breakpoint coverage, a dual-clustering strategy was implemented. Chimeric read pairs, in which one read aligns to HBV and the second paired-read aligns to GRCh38, were extracted from the BAM alignment file and processed using bedtools bamtobed[17] in paired-end mode. Integration junction reads, in which one read was composed of both HBV and hg38 sequence, were split and treated as pseudo-read pairs and otherwise processed identically. Read pairs which aligned to more than 3 loci were discarded. This approach yielded specific breakpoint coordinates while still maintaining almost all reads deriving from chimeric nucleic acid fragments. Breakpoint read clusters were generated from processed chimeric reads using bedtools cluster with a window size of 500 bp. Clusters were defined by HBV coordinate, human coordinate, and the strand of reads supporting the cluster.

HBV genome coverage for both DNA and RNA samples was calculated using bedtools genomecov using all reads which aligned to the GRCh38:HBV hybrid genome with mapping quality > 20 and the primary read alignment flag. For visual comparisons, read depth for each sample was normalized to the maximum HBV genome depth of coverage.

### Estimation of HBV integration frequency

**Fig 1:**
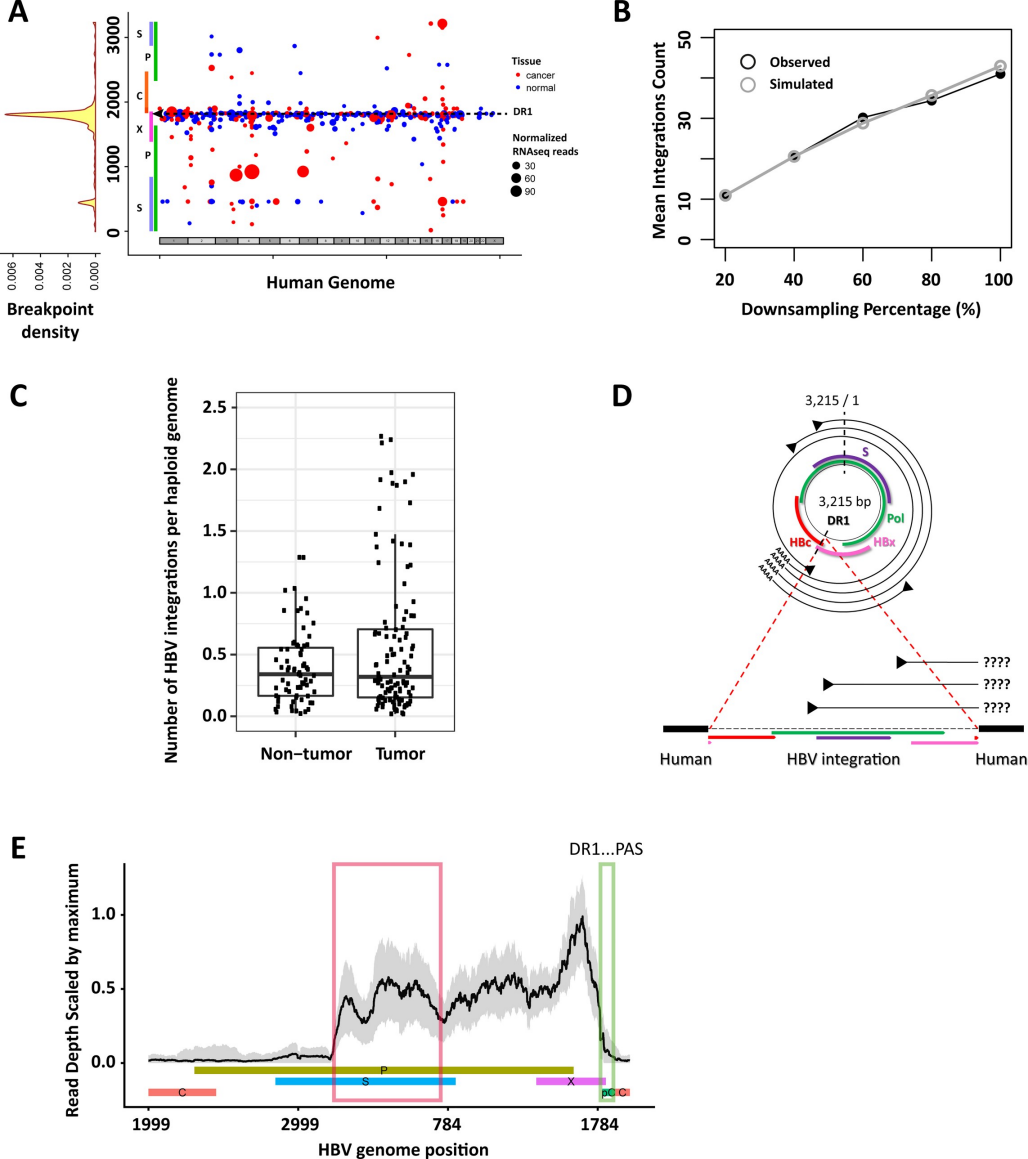
Estimation of hepatitis B integration frequency and its contribution to the viral S antigen expression. (**A**) The hot-spot of viral-human fusion junctions on the viral genome. Although viral integrations on the human genome tend to spread across every chromosome (x-axis), the DR1 region seems to be the most commonly used fusion breakpoint in the HBV genome. Each dot represents a viral integration identified in the liver RNAseq data. The red dots represent data from tumor samples while the blue dots represent data from non-tumor samples. Size of dot is proportional to the normalized read count supporting each integration call. The read count is normalized to 1 million library reads. (**B**) The number of HBV DNA integrations as a function of sequencing-depth coverage: real data (black) versus result from a Binomial modeling (grey). (**C**) HBV integration frequency representing the number of viral integration per sequenced haploid genome in non-tumor and tumor samples. (**D**) Schematic of HBV genome structure, four sets of transcripts, and linearization during integration into the human genome. Most HBV integrations fused to the human genome at the DR1 region downregulate the viral expression of the DR1-PAS region and the core gene (green box and gene labeled C in panel E). (**E**) Aggregated RNAseq coverage across all non-tumor samples (n = 59) on the HBV genome (x-axis). The median value was shown as a solid line with grey bars indicating the variance. Coverage is scaled to one across all samples for comparison purposes. The depth of RNAseq coverage drops right after the DR1 locus. The expression level of DR1-PAS window (green box) represents the transcription from circular genome while the expression of the S window (red box) represents a total HBs expression from both the circular genome and integrated linear HBV genome. The core gene (labeled C) expression was depleted in the integrated HBV. PAS—viral PolyA signal site.

During a typical Next Generation Sequencing (NGS) workflow, genomic DNA from a sample with a large number (typically 200,000–1,000,000) of cells is extracted and fragmented, followed by amplification and sequencing (i.e. by Illumina technology). This sequencing workflow ends once the read depth coverage reaches a predefined level. In a typical whole genome sequencing (WGS) study, 30x coverage is often considered sufficient for calling germline variants in the diploid human genome. However, even much higher coverages than 30x would be insufficient to capture all possible HBV viral integrations across hundreds of thousands of cells, since viral integrations appear to be distributed randomly across the human genome and generate a heterogeneous integration sites across hepatocytes [12] (Fig 1A). Because the heterogeneity of the viral integration alleles at the cell-level make it impossible to recover all integrations from limited depth of coverage of sequencing, we therefore attempted to indirectly estimate the frequency of independent HBV integration events as follows: Let *I* be the number of genomes (content of DNA extraction) in a given sample and let *J* be the number of genomic locations (length of the human genome; ~3.2 billion base pairs). For a given genome *i* and a given genomic location *j*, let *x*_*ij*_ *= 1* if an HBV integration is present and *x*_*ij*_ *= 0* if an HBV integration is absent. By definition, the total number of HBV integrations across all genomic locations in all genomes in the human tissue sample therefore is ∑_*i*_∑_*j*_*x*_*ij*_, and the HBV integration frequency, i.e. the average number of HBV integrations per genome, is $\frac{\sum_{i}\sum_{j}x_{ij}}{I}$.

Let *Y*_*j*_ be the number of observed HBV integrations at a given genomic location *j* after sequencing the processed sample. If we assume that *Y*_*j*_ approximately follows a binomial distribution, *Y*_*j*_ *~ binomial(n*_*j*_, *p*_*j*_*)*, where $p_{j}=\frac{\sum_{i}x_{ij}}{I}$ is the proportion of true HBV integrations at genomic location *j* and *n*_*j*_ is the read depth at genomic location *j*, then the maximum likelihood estimate (MLE) of the proportion of true HBV integrations at genomic location *j* is ${\hat{p}}_{j}=\frac{y_{j}}{n_{j}}$, where *y*_*j*_ is the observed number of HBV integrations at genomic location *j*. The MLE of the HBV integration frequency, i.e. the average number of HBV integrations per genome, is then $\hat{y}=\sum_{j}{\hat{p}}_{j}$. To validate our assumptions, we performed down-sampling of a high-coverage patient sample and found that the mean integration count matched our model well (Fig 1B).

As it is unknown whether an observed HBV integration is due to an independent viral integration event or due to clonal expansion following an integration, we conservatively estimated the number of unique HBV integration events at each genomic location *j* as a lower bound estimate of ${\hat{p}}_{j}$. This conservative estimate can be denoted as: ${\hat{\theta }}_{j}=\frac{I[y_{j}]}{n_{j}}$, where $I[y_{j}]=\left\{ \begin{array}{c} 0,\;if\;y_{j}=0\\ 1,\;if\;y_{j}>0 \end{array}\right.$, and serves as a lower bound because: ${\hat{p}}_{j}=\frac{y_{j}}{n_{j}}\geq \frac{I[y_{j}]}{n_{j}}={\hat{\theta }}_{j}$. The corresponding lower bound estimate of the HBV integration frequency would then be: $\sum_{j}{\hat{\theta }}_{j}$. When estimating the average number of HBV integrations, we set *n*_*j*_ to be *n*, which approximates read depth of coverage at each genomic position to be the genome-wide average depth of coverage.

There are two important benefits worth noting about our approach. First, our method does not require knowing the number of cells put into the WGS workflow. We derive that sequencing a sample to, for example, 30x coverage, is equivalent to sampling 30 genomes from however many cells used as an input for the sequencing library preparation. Second, our primary goal was to identify the frequency of independent integration events, not the total number of viral integrations. Therefore, it is critical in our estimation to count for the increase of HBV integration burden due to subsequent hepatocyte clonal expansion. If a clonal expansion occurs and happens to carry a specific integration, this integration will share identical genomic locus across expanded hepatocytes. In our estimation, this event is counted once and only once because there was only a single founder integration event prior to the subsequent clonal expansion. The increase of viral integration burden due to a clonal expansion is not directly related to the viral replication process, therefore it should not be considered as independent integration events.

## Results and discussion

We examined the paired-end whole genome sequencing data from 72 non-tumor samples in HBV positive HCC patients reported in recent studies [10, 14]. Of these 72 patients, 53 were infected with HBV genotype C and 19 with HBV genotype B. We estimated the average HBV integration frequency to be 0.431 per haploid genome (median = 0.347, min = 0.022, max = 2.670; Fig 1C). We did not detect any HBV genotype specific bias (mean integration rate *i*_*GT B*_ = 0.434 and *i*_*GT C*_ = 0.422, Wilcoxon rank sum test *p* = 0.41, S1 Fig). Given a typical diploid cell configuration, this translates to an average frequency of 0.844 per diploid genome. Since we counted only unique viral integration events, this represents the lower-bound estimation of the actual viral integration burden (see Methods). Although this estimate is very conservative, the order of magnitude is rather high, indicating that HBV integration in CHB patients is potentially very pervasive and higher than previously thought[1, 18]. Since this analysis is based on bulk sequencing data, this lower bounds estimate represents the average viral integration frequency throughout HBV infected liver tissue sample and is not characterizing individual cells. It is not possible to discern whether this estimate is a result of a few cells harboring many distinct integrations or large number of cells each harboring few integration events. Future single cell sequencing based study is necessary to define the spatial distribution of viral integrations in CHB patients.

This estimation method can be applied to tumor samples as well. Since only independent viral integrations with distinct genomic location are included in the frequency calculation, our method inherently controls possible over-estimation of viral integration due to clonal expansion, a phenomenon characteristic of tumor evolution. The average frequency of HBV integration across 120 tumor tissue samples was 0.674 per haploid genome (median = 0.340, min = 0.019, max = 6.005). Interestingly, integration estimates from both tumor and non-tumor tissue samples are comparable suggesting that viral integration frequency is relatively constant regardless of oncogenic progression. To confirm that none of the detected integrations in the liver tissue samples were a result of PCR or mapping artifacts, DNA sequencing data from matched blood samples was analyzed as a negative control using the same methodology. We did not detect any HBV integrations in the blood samples suggesting that our results across both tumor and non-tumor tissue samples reflect true viral integration phenomenon.

Given the prolific rate of HBV integration in non-tumor liver tissue, we further quantified the relative contribution of integrated HBV genomes to HBsAg RNA expression. Despite alternate transcription start sites, all four major families of cccDNA transcripts share a common 3’ polyadenylation signal locus [19, 20] (Fig 1D). Transcripts generated from the integrated HBV DNA template, however, tend to be disrupted at the DR1 locus, a common integration junction on the HBV genome (Fig 1A and 1D). Since the majority of expressed HBV DNA integrations are fused with the human genome across the DR1 locus (Fig 1A) and lead to a disruption of the DR1-PAS region, any transcriptional activity of the DR1-PAS region (designated as Depth_DR1-PAS_) should originate primarily from circular viral template DNA (cccDNA). Conversely, transcripts covering the S gene (Depth_S_) would originate from either cccDNA or integrated linear HBV DNA. Following this rationale, we estimated the proportion of transcripts originating from integrated HBV using the formula: (Depth_S_—Depth_DR1-PAS_)/Depth_S_ (Fig 1E). Using this calculation, we determined that the median percentage of transcripts originating from integrated HBV DNA in tumor samples was 85.5% and 77.2% in non-tumor samples (median of pooled tumor and non-tumor was 82.8%). The difference between tumor and non-tumor samples was not statistically significant (Wilcoxon rank sum test, *p* = 0.72). This observation is comparable to Wooddell et al [9] findings (66.4%), which implicated a significant contribution of integrated HBV to serum HBsAg levels in CHB patients. Because DNA and RNA extraction did not sample the same cells, further investigation comparing HBV integrations from DNAseq and RNAseq directly is not adequate.

Together, these results highlight the impact of integrated HBV DNA in CHB patients when developing HBV therapeutic endpoints. With an average HBV DNA integration rate in non-tumor tissue samples of 0.844 integrations per diploid genome, integrated HBV would be expected to exert a dominant HBsAg transcriptional burden in patients–a hypothesis supported by the finding that ~80% of HBs transcripts are derived from integrated HBV DNA. Although we have restricted our analyses to conservative estimates of integration frequency and transcript proportion, there are additional caveats which should be highlighted. First, due to limitations in sample availability, we do not know the extent to which the integration profile of non-tumor tissue samples from HCC patients diverge from that of liver tissue of CHB patients. Second, sequenced bulk liver tissue samples will be composed of a heterogeneous mix of cell types. While we expect that only hepatocytes would be infected by HBV, DNA from all tissue resident cell types was sequenced, which would lead to an under-estimation of viral integration rate using our method. Third, HBV transcripts could undergo degradation of the 3’ end near the polyadenylation tail, leading to fewer reads spanning the DR1-PAS region and possible overestimation of HBsAg transcription from integrated HBV DNA template. This, however, doesn’t seem to be the case as analysis of human gene RNA-seq coverage did not revealed 3’-end degradation or 5’-end bias.

Additionally, we do not account for integration sites outside the DR1-PAS region when estimating relative contribution of integrated DNA to transcriptional activity. Given that the DR1 site has been described as the major junction for viral integrations, our expectation would be that RNA originating from the fraction of non-DR1-PAS integration sites would not substantially impact our findings.

We consider this work to have two main implications for the long-term development of HBV-directed therapies. First, our work supports the observation that integrated HBV DNA serves as a template for HBs antigen transcription and implies that quantitative serum level of HBsAg is a confounded biomarker when evaluating disease progression in CHB patients. Based on the findings of our work and others[21], HBc gene expression would be expected to reflect HBV viral replication activity more accurately. Combination of therapeutics targeting infected cells with either cccDNA or integrated viral DNA will be needed to eliminate HBsAg levels and increase HBsAg seroconversion rates. Our results emphasize the significance of various strategies that are currently being tested including RNAi, targeted DNA editing technologies [9, 22, 23], and the modulation of the host immune system via therapeutic vaccine or immune checkpoint blockades[24, 25]. Second, CHB patients with normal alanine aminotransferase (ALT) levels are generally not treated with interventional therapies[26]. We would like to raise the awareness that patients with normal ALT levels may still be exposed to the risk associated with constitutive viral integrations. The accumulation of HBV integrations in the human genome imposes a mutation burden by disrupting important regulatory genes, drives aberrant gene expression by fusion of human-viral sequences, and induces genomic rearrangements[5], all of which pose the risk of oncogenic transformation. Indeed, a recent study on non-cirrhotic CHB patients with normal to minimally elevated ALT demonstrated that patients receiving antiviral therapy had reduced risk of liver cancer compared to an untreated cohort [27].

In summary, we observed that genomic integration of HBV DNA occurs frequently in non-tumor liver tissue of HBV infected HCC human subjects, and that integrated HBV DNA is the primary contributor to the HBsAg transcriptional burden. These findings highlight the need to re-visit the clinical interpretation of HBsAg levels of CHB patients and to determine the increased oncogenic risk to untreated CHB patients caused by viral integrations.

## Supporting information

S1 Fig HBV integration frequency across HBV genotypes. The median HBV integration frequencies across HBV genotypes B and C are 0.434 and 0.422, respectively. This difference is not statistically significant (Wilcoxon rank sum test, *p* = 0.41).
(TIF)

S1 Table Origin of samples used in this study. (DOCX)
