# Supplementary figures and images for: Genomic modeling of hepatitis B virus integration frequency in the human genome
Source: PLoS One. 2019 Jul 29;14(7):e0220376. doi: 10.1371/journal.pone.0220376 (PMC6663024; doi:10.1371/journal.pone.0220376)

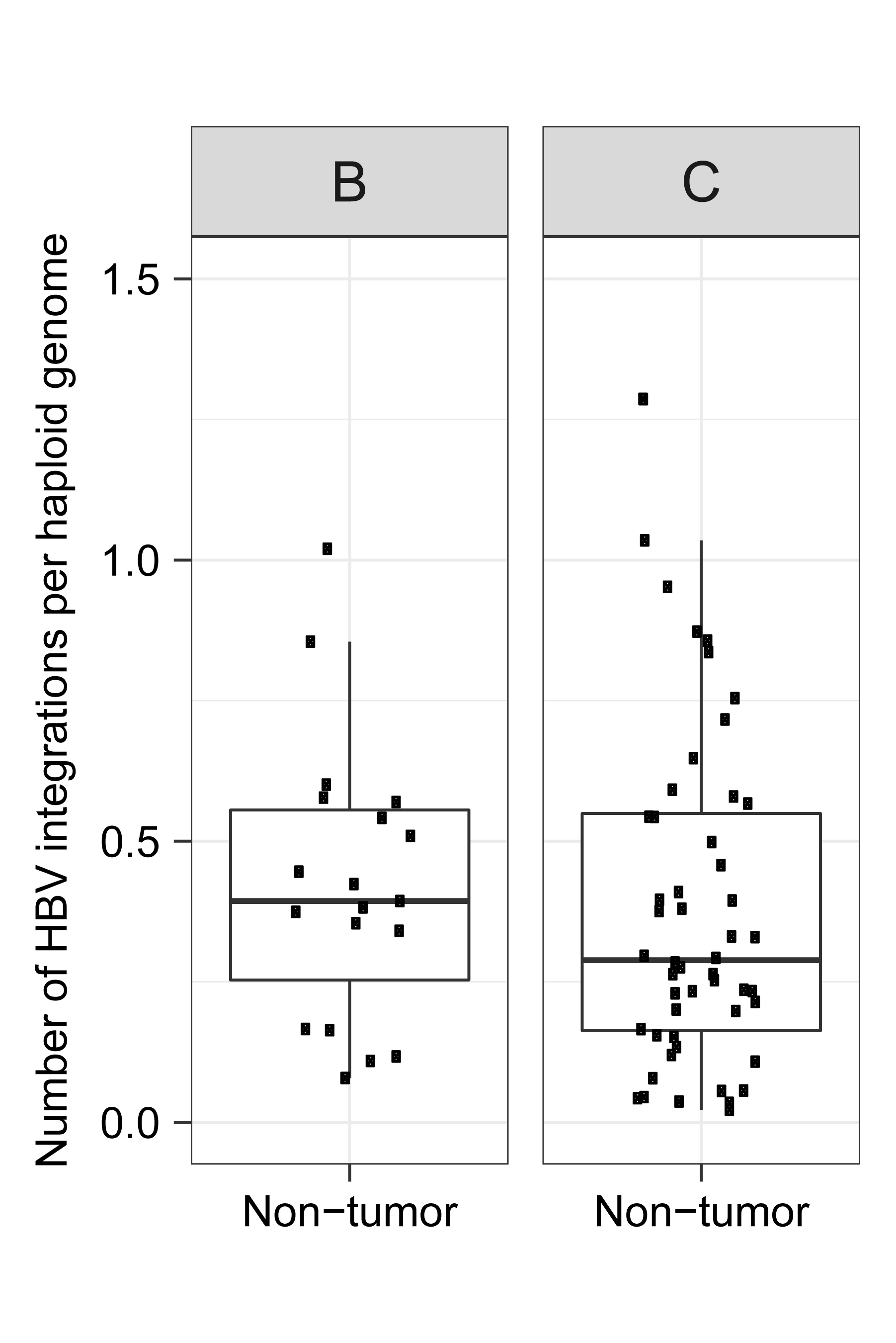

Supplement: S1 Fig — The median HBV integration frequencies across HBV genotypes B and C are 0.434 and 0.422, respectively. This difference is not statistically significant (Wilcoxon rank sum test, p = 0.41). (TIF) [file pone.0220376.s001.tif]
