# Supplementary material for: Genomic modeling of hepatitis B virus integration frequency in the human genome
Source: PLoS One. 2019 Jul 29;14(7):e0220376. doi: 10.1371/journal.pone.0220376 (PMC6663024; doi:10.1371/journal.pone.0220376)
Supplement: S1 Table — (DOCX) [file pone.0220376.s002.docx]

**Supporting information**

**S1 Table. Origin of samples used in this study.**

| **Nucleic Acid** | **Tissue** | **Study** | |
| --- | --- | --- | --- |
|  |  | Fujimoto et al. ^10^ | Sung et al. ^14^ |
| **DNA** | tumor | 43 | 77 |
|  | adjacent non-tumor | 0* | 72 |
| **RNA** | tumor | 30 | NA |
|  | adjacent non-tumor | 30 | NA |
